# MicroRNA-92a-3p Inhibits Cell Proliferation and Invasion by Regulating the Transcription Factor 21/Steroidogenic Factor 1 Axis in Endometriosis

**DOI:** 10.1007/s43032-021-00734-9

**Published:** 2023-01-17

**Authors:** Jingwen Zhu, Zijin Xu, Peili Wu, Cheng Zeng, Chao Peng, Yingfang Zhou, Qing Xue

**Affiliations:** 1 Department of Obstetrics and Gynecology, Peking University First Hospital, No.1 Xi’anmen Street, Beijing, 100034 China; 2 Department of Reproductive Medicine, Key Laboratory for Major Obstetric Diseases of Guangdong Province, and Key Laboratory for Reproduction and Genetics of Guangdong Higher Education Institutes, the Third Affiliated Hospital of Guangzhou Medical University, Guangzhou, China

**Keywords:** Endometriosis, miR-92a-3p, TCF21, SF-1

## Abstract

Endometriosis (EMS) is an estrogen-dependent disease. However, little is known about the regulation of estrogen, a potential therapeutic target, in EMS, which remains very poorly managed in the clinic. We hypothesized that microRNAs (miRNAs) can be exploited therapeutically to regulate transcription factor 21 (TCF21) and steroidogenic factor-1 (SF-1) gene expression. In our study, paired eutopic and ectopic endometrial samples were obtained from women with EMS and processed by a standard protocol to obtain human endometrial stromal cells (EMs) for in vitro studies. We found that miR-92a-3p levels were decreased in ectopic endometrium and ectopic stromal cells (ESCs) compared with paired eutopic lesions. miR-92a-3p overexpression significantly suppressed the proliferation and migration of ESCs, whereas a decreased level of miR-92a-3p generated the opposite results. Next, we identified TCF21 as a candidate target gene of miR-92a-3p. In vitro cell experiments showed that miR-92a-3p negatively regulated the expression of TCF21 and its downstream target gene SF-1. Moreover, cell proliferation and invasion ability decreased after the silencing of SF-1 and increased after SF-1 overexpression. We also observed that silencing SF-1 while inhibiting miR-92a-3p partially blocked the increase in cell proliferation and invasion ability caused by miR-92a-3p knockdown while overexpressing both SF-1 and miR-92a-3p mitigated the impairment in cell proliferation and invasion ability caused by miR-92a-3p overexpression. Our results may provide a novel potential therapeutic target for the treatment of EMS.

## Introduction

Endometriosis (EMS) is a chronic and estrogen-dependent gynecological disorder that is characterized by the presence of endometrial-like tissue in aberrant locations outside the uterus [1]. Approximately 10% of reproductive-aged women suffer from this condition, which is often accompanied by chronic pelvic pain and subfertility [2]. Although the pathogenesis of EMS is not well known, it is clear that alterations in estrogen signaling play an important role in the development and progression of the disease [3].

Steroidogenic factor-1 (SF-1), which is encoded by NR5A1, is a member of orphan nuclear receptor (NR) superfamily 5, and it binds to a specific response element named the nuclear receptor half-site in the promoters of several steroidogenic genes [4]. Previous studies have reported that while NR5A1 expression is barely detectable in the eutopic endometrium, its mRNA level is approximately 12,000-fold higher in ectopic lesions [5]. In endometriotic stromal cells, NR5A1 serves as the key transcription factor that transactivates the promoters of both steroidogenic acute regulatory protein (StAR) and aromatase (encoded by the CYP19A1 gene), which leads to increased estradiol formation [6, 7]. Independent of its steroidogenic actions, SF-1 promotes the proliferation and invasion of adrenocortical carcinoma cells. [8, 9] However, in EMS, the role of SF-1 in the regulation of functional changes in ectopic endometrial cells has not been reported to date.

Transcription factor 21 (TCF21) is a member of the class II bHLH transcription factor superfamily and is widely expressed in multiple tissues and organs [10]. TCF21 is highly expressed in endometriotic stromal cells compared with paired eutopic stromal cells [11]. The overexpression of TCF21 promotes the proliferation and invasion ability of ectopic stromal cells (ESCs). Moreover, Ganieva et al. found that TCF21 might be a key regulator of the inactivation of periostin, which can cause fibrosis in endometriotic lesions [12]. However, the mechanism underlying the high TCF21 expression in EMS is not fully understood.

miRNAs are small noncoding RNAs that control gene expression at the post-transcriptional level via the immediate inhibition of target mRNA [13]. Emerging evidence indicates that the deregulation of miRNAs is involved in EMS [14]. Aberrant expression of miR-92a-3p has been detected in multiple cancers, and miR-92a-3p has been found to be aberrantly expressed in EMS [15–17]. Thus, we aimed to determine the functional roles of miR-92a-3p in EMS development.

In this study, we hypothesized that miR-92a-3p may be involved in the pathogenesis of EMS by controlling ESC invasiveness and proliferation via the regulation of TCF21 and SF-1 expression.

## Materials and Methods

### Patients and Primary Cell Culture

In total, 11 patients aged 20–35 years with regular menstrual cycles and no history of hormonal treatment for at least 3 months before surgery were enrolled in the study. All were determined to be in the proliferative phase of their menstrual cycle, as assessed by the timing of their last menstrual period and histological analysis of the endometrium. Eleven self-controlled pairs of ectopic endometrial tissues from the cyst walls of ovarian endometriomas and eutopic endometrial tissues were obtained from women who underwent laparoscopic excision of ovarian EMS with hysterectomy. Informed consent was obtained from all patients using protocols approved by the Institutional Review Board of Peking University (No. 2020 [280]). Human endometrial stromal cells (EMs) and ESCs were isolated from the collected tissues using a protocol previously described by Ryan et al. with minor modifications [18]. In brief, tissues were washed with phosphate-buffered saline (PBS) and minced into small (1 mm^3^) pieces. After enzymatic digestion of the minced tissues with collagenase (1 mg/mL) (Sigma-Aldrich, St. Louis, MO, USA) and DNase (0.04 mg/mL) (Sigma-Aldrich) for 1 h at 37 °C, the dissociated tissues were sequentially filtered through 70-μm and 20-μm nylon mesh to remove the epithelial cells. The filtered cells were centrifuged; suspended in Dulbecco’s modified Eagle’s medium (DMEM)/F12 (1:1) (HyClone, Logan, UT, USA) supplemented with 10% (v/v) fetal bovine serum (FBS; Gibco/BRL, Grand Island, NY, USA), 100 U/mL penicillin (Lonza, Basel, Switzerland), 100 U/mL streptomycin (Lonza), and 250 ng/mL amphotericin B (Lonza); and incubated at 37 °C in a humidified atmosphere containing 5% CO2. Stromal cells were plated and allowed to adhere to plastic dishes for approximately 30 min, after which blood cells and debris were removed by rinsing with PBS. The purity of the stromal cells was > 98%, as determined by positive cellular staining for vimentin, a specific marker of stromal cells. All experiments were conducted before passage 3.

### Next-Generation RNA Sequencing

Five paired ectopic and eutopic endometrial tissues were selected by simple random sampling for RNA sequencing. The samples were sequenced using the Illumina HiSeq 2000/2500 platform, and 50-bp single-end reads were generated. Raw data (raw reads) in fastq format were first processed through custom Perl and python scripts. In this step, clean data (clean reads) were obtained by removing reads containing poly-N, with 5’ adapter contaminants, without a 3’ adapter or insert tag, or containing poly-A/T/G/C and low-quality reads from the raw data. At the same time, the Q20, Q30, and GC content of the raw data were calculated. Then, a certain range of lengths of clean reads was chosen to perform all downstream analyses. Sequencing and data collection were conducted by Novogene Bioinformatics Technology Co., Ltd. (Beijing, China).

### RNA Extraction and Quantitative Analysis by Quantitative Real-Time Polymerase Chain Reaction (qRT-PCR)

Total RNA was extracted from ESCs and EMs using TRIzol reagent (Invitrogen, Carlsbad, California) according to the manufacturer’s protocol. For reverse transcription, cDNA was generated using an ABI High-Capacity cDNA Archive Kit (Applied Biosystems, Foster City, California) or TransScript Green miRNA Two-Step qRT-PCR SuperMix Kit (Trans, Beijing, China). qRT-PCR was conducted using an ABI 7500 sequence detection system with SYBR Green to determine the RNA expression levels of the genes of interest. Expression levels were normalized to the expression of GAPDH (mRNA) or U6 RNA (miRNA). The primers were as follows: SF-1, 5’-GGGTGAAGCCACCGTCATC-3’ and 5’-CTTCTTATCCCCC AAGTCCTCAGT-3’; GAPDH, 5’-GAAGGTGAAGGTCGGAGTC-3’ and 5’-GA AGATGGTGATGGGATTTC-3’; TCF21, 5’-AGCTACATCGCCCACTTGAG-3’ and 5’-CGGTCACCACTTCTTTCAGG-3’; miR-92a-3p, 5’-TATTGCACTTGT CCCGGCC-3’; miR-25-3p, 5’-CCTGTGGGCCACCTAGTCAC-3’; miR-363-3p, 5’-AGGGGCTGGCTTTCCTCT-3’; and U6, 5’-AGAGAAGATTAGCATGGC CCTG-3’.

### Western Blotting

Total proteins were obtained from tissues and cells lysed in RIPA buffer (KeyGen Biotech, Nanjing, China) supplemented with a protease inhibitor cocktail (Amresco, Solon, Ohio) and phosphatase inhibitor (KeyGen Biotech). The protein content was quantified using a BCA assay kit (KeyGen Biotech). Equal amounts of protein were subjected to 10% SDS-PAGE for electrophoresis and transferred to nitrocellulose membranes (Millipore, Bedford, MA, USA) with the use of a Transblot apparatus (Bio-Rad, Hercules, CA, USA). The membranes were blocked at room temperature for 1 h with 5% bovine serum albumin and then incubated with anti-TCF21 antibody (ab182134, 1:1000 dilution; Abcam), anti-SF-1 antibody (ab168380, 1:1000 dilution; Abcam), and anti-GAPDH antibody (1:1000 dilution; ZSGB-BIO, Beijing, China) at 4 °C overnight. The next day, the membranes were incubated with the appropriate secondary antibodies at a 1:5000 dilution for 1 h at 37 °C. Protein bands were visualized by enhanced chemiluminescence (ECL) solution (Syngene). The intensities of the western blot bands were analyzed using ImageJ software and normalized with respect to GAPDH. Independent experiments were performed using cultured cells from at least three different subjects and repeated three times.

### Cell Transfection

ESCs were cultured to approximately 80–90% confluence at the time of transfection. Empty pENTER plasmid, pENTER-NR5A1 plasmid (Vigene Biosciences), hsa-miR-92a-3p mimics, hsa-miR-92a-3p inhibitor or hsa-miR-negative control (Guangzhou RiboBio Co., Ltd., Guangzhou, China) were transfected into ESCs using Lipofectamine 3000 transfection reagent (Invitrogen, Carlsbad, CA) according to the manufacturer’s protocol. After being cultured to approximately 70–80% confluence, ESCs were transfected with either a nontargeting negative control siRNA (Invitrogen) or siRNAs against human SF-1 (Invitrogen) at 100 nmol/L using Lipofectamine RNAiMAX (Invitrogen) in Opti-MEM-reduced serum medium (Invitrogen). After 24 or 48 h of incubation, the cells were harvested for RNA or protein isolation.

### Cell Counting Kit-8 (CCK-8) Assay

The CCK-8 assay was carried out according to the manufacturer’s instructions (Dojindo Molecular Technologies, Xiongben, Japan). Briefly, 24 h after transfection, ESCs were digested and seeded into a 96-well plate at a density of 3000 cells/well and then incubated at 37 °C for 4 days. The cells were incubated with 100 μL of DMEM/F12 containing 10 μL of CCK-8 reagent at 37 °C for 4 h, and the absorbance of each well was then measured at a wavelength of 450 nm using a microplate reader. Independent experiments were performed using cultured cells from three different subjects and repeated three times.

### Matrigel Invasion Assay

In vitro invasion assays were performed using Matrigel-coated (1:8 dilution) 24-well Transwell chambers (8-μm pore size, 6.5-mm diameter, Corning, USA). Next, 2 × 10^5^ siRNA-transfected or plasmid-transfected primary ESCs were plated in the upper chamber in serum-free media. The lower chamber was filled with 600 μL of DMEM/F12 containing 20% FBS. The cells were then incubated at 37 °C for 48 h. The cells on the upper surface of the filter together with Matrigel were removed by wiping with a cotton swab. The inserts were then fixed in methanol for 30 min at room temperature and stained with hematoxylin. The stained cells reaching the lower surface were observed and imaged using an Olympus DP71 microscope (Olympus, Tokyo, Japan). Five randomly selected fields were quantified for each experiment. Independent experiments were performed using cultured cells from three different subjects and repeated three times.

### Statistical Analyses

All experiments were performed at least three times. Comparisons between groups were performed using the two-tailed Student’s *t*-test. Comparisons among more than two groups were performed using one-way analysis of variance (ANOVA). All values are shown as the means ± standard error of the mean, and a *P* value of < 0.05 was considered significant. Statistically significant differences are shown as follows: ***P < 0.001, **P < 0.01, and *P < 0.05.

## Results

### The Expression of miR-92a-3p Is Downregulated in Human Endometriotic Tissues

**Fig. 1:**
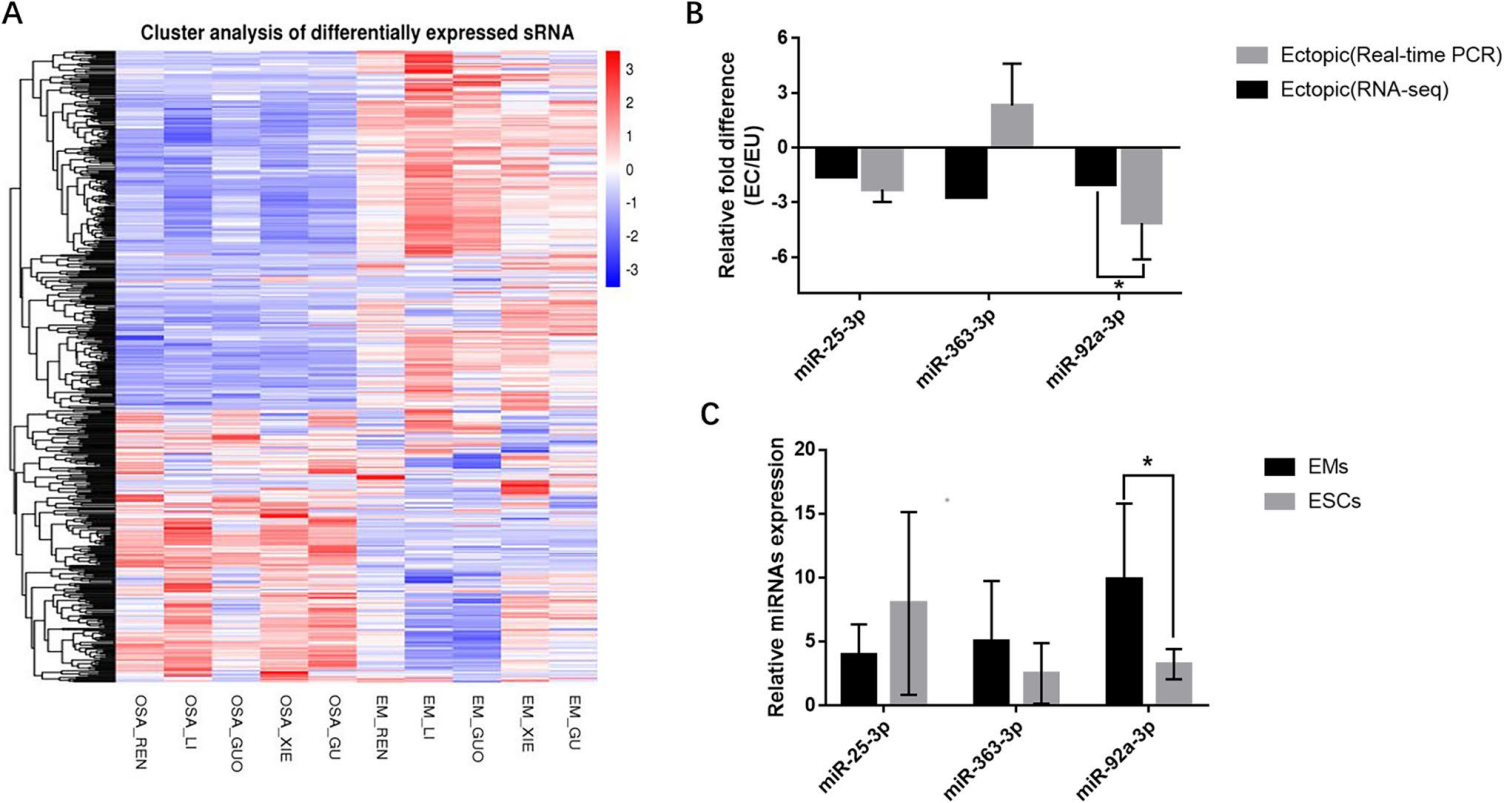
The expression of miR-92a-3p is downregulated in human endometriotic tissues. (**a**) miRNA expression profiles of paired eutopic and ectopic endometrium of endometriosis patients. Red indicates relatively high expression, and blue indicates relatively low expression. (**b**) Total RNA was extracted from paired endometriotic and endometrial tissues. miRNA levels were measured by RT-PCR (*n* = 5; *, *P* < 0.05, *t*-test). (**c**) Human primary EMs and ESCs were isolated, and miRNA expression levels were measured by RT-PCR (*n* = 7; *, *P* < 0.05, *t*-test)

In this study, we analyzed eutopic and ectopic endometrial samples from women with EMS by RNA sequencing. The expression levels of a total of 402 miRNAs (comprising 172 upregulated miRNAs and 230 downregulated miRNAs) were found to differ significantly (≥ twofold change) between ectopic and eutopic endometrium (Fig. 1a). Among them, members of the miR-92a family, including miR-25-3p, miR-92a-3p, and miR-363-3p, showed downregulated expression in endometriotic tissues. To confirm the altered expression of the miR-92a family in EMS, we tested the expression of miR-25-3p, miR-92a-3p, and miR-363-3p in paired eutopic and ectopic tissues using qRT-PCR. In agreement with the RNA-seq results, miR-25-3p and miR-92a-3p expressions were significantly lower in endometriotic lesions than in their paired endometrium. However, there were no significant differences in miR-363-3p levels between ectopic and eutopic tissues (Fig. 1b). Moreover, only miR-92a-3p expression was significantly decreased in ESCs compared with eutopic stromal cells (Fig. 1c).

### miR-92a-3p Inhibited the Proliferation and Invasion of ESCs

**Fig. 2:**
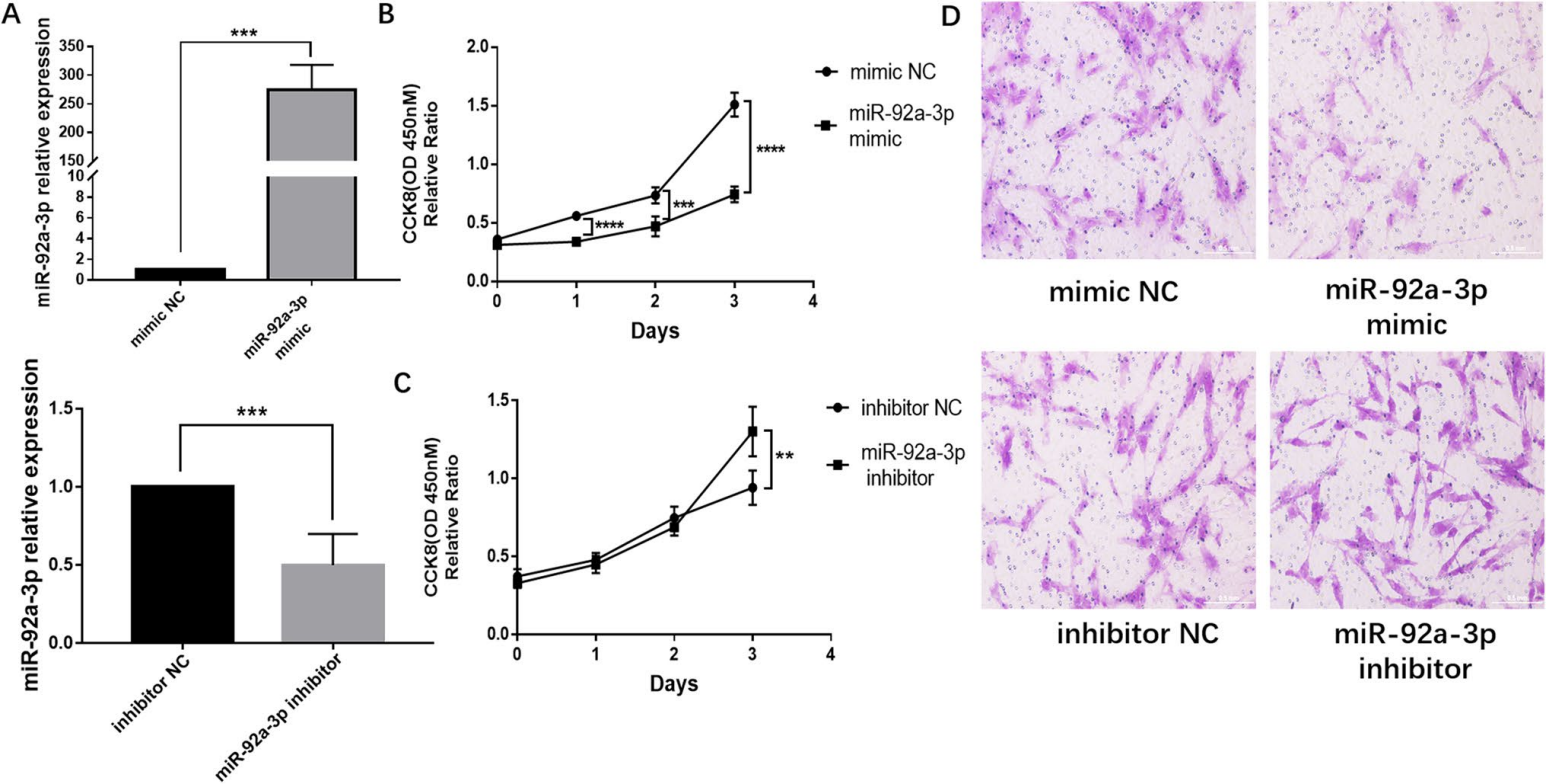
miR-92a-3p inhibited the proliferation and invasion of ESCs. (**a**) ESCs were transfected with the indicated mimic or inhibitor for 48 h and harvested for real-time quantitative PCR to verify the transfection efficiency (*n* = 3; ***, *P* < 0.001, *t*-test). (**b**) CCK-8 assay showed that miR-92a-3p overexpression inhibited endometrial cell proliferation (*n* = 3; ****, *P* < 0.0001; ***, *P* < 0.0001, ANOVA). (**c**) CCK-8 assay showed that miR-92a-3p downregulation promoted endometrial cell proliferation (*n* = 3; **, *P* < 0.01, ANOVA). (**d**) At 48 h post-transfection with the indicated mimic or inhibitor, the invasion of ESCs was assessed using Matrigel invasion chambers (*n* = 3). Magnification, 40 ×

To fully understand the role of miR-92a-3p in EMS progression, we transfected primary ESCs with a miR-92a-3p mimic or inhibitor, and functional studies of cell proliferation and invasion were performed with these cells. qRT-PCR was used to verify the miR-92a-3p transfection efficiency (Fig. 2a). As presented in Fig. 2b, the CCK-8 assay indicated that the proliferation of ESCs was lower in cells transfected with the miR-92a-3p mimic than in those transfected with miR-92a-3p inhibitor. In contrast, relative cell proliferation ability was increased after miR-92a-3p inhibitor transfection (Fig. 2c). Moreover, the Transwell invasion assays showed that the invasion process of ESCs was significantly impaired in the presence of miR-92a-3p mimics (Fig. 2d), while miR-92a-3p inhibitors significantly promoted ESC invasion (Fig. 2d). Collectively, these findings suggest that the downregulation of miR-92a-3p in ESCs is responsible for the increased proliferation and invasion of these cells in EMS.

### miR-92a-3p Regulated TCF21 and SF-1 Expression in ESCs

**Fig. 3:**
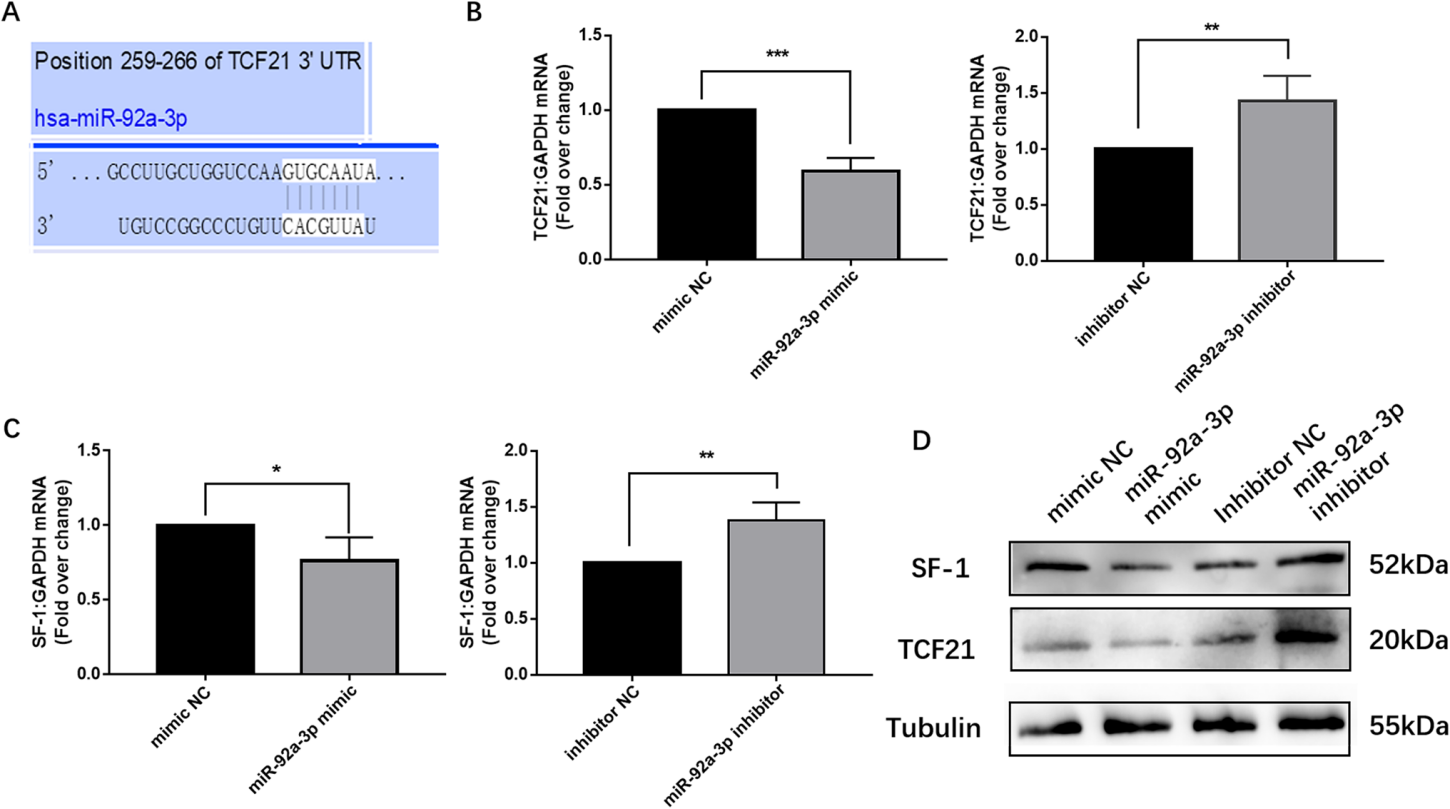
miR-92a-3p regulated TCF21 and SF-1 expression in ESCs. (**a**) The predicted miR-92a-3p binding sites of the TCF21 3’UTR. (**b** and **c**). ESCs were transfected with the indicated mimic or inhibitor for 48 h and harvested for real-time RT-PCR (*n* = 3; ***, *P* < 0.001, **, *P* < 0.01, *, *P* < 0.05; *t*-test). (**d**) ESCs were transfected with the indicated mimic or inhibitor for 48 h. Cells were harvested for western blotting performed with the indicated antibodies (*n* = 3)

Multiple algorithms (starBase, TargetScan, miRDB) were used to identify the candidate targets of miR-92a-3p. As a result, TCF21 was predicted to be the most likely target of miR-92a-3p. Figure 3a shows the alignment between miR-92a-3p and a highly conserved region within the 3’UTR of human TCF21, which represents a putative target sequence that can confer translational inhibition by miR-92a-3p. Then, we examined the impact of miR-92a-3p level on the expression of its predicted target gene TCF21 and the downstream gene SF-1. As expected, miR-92a-3p mimic transfection decreased the mRNA and protein levels of TCF21 and SF-1 in ESCs, while miR-92a-3p inhibitor transfection increased the mRNA and protein levels of TCF21 and SF-1 in these cells (Figs. 3b and c). Altogether, our results indicate the ability of miR-92a-3p to regulate TCF21 and SF-1 expression in ESCs.

### SF-1 Promoted the Proliferation and Invasion of ESCs

**Fig. 4:**
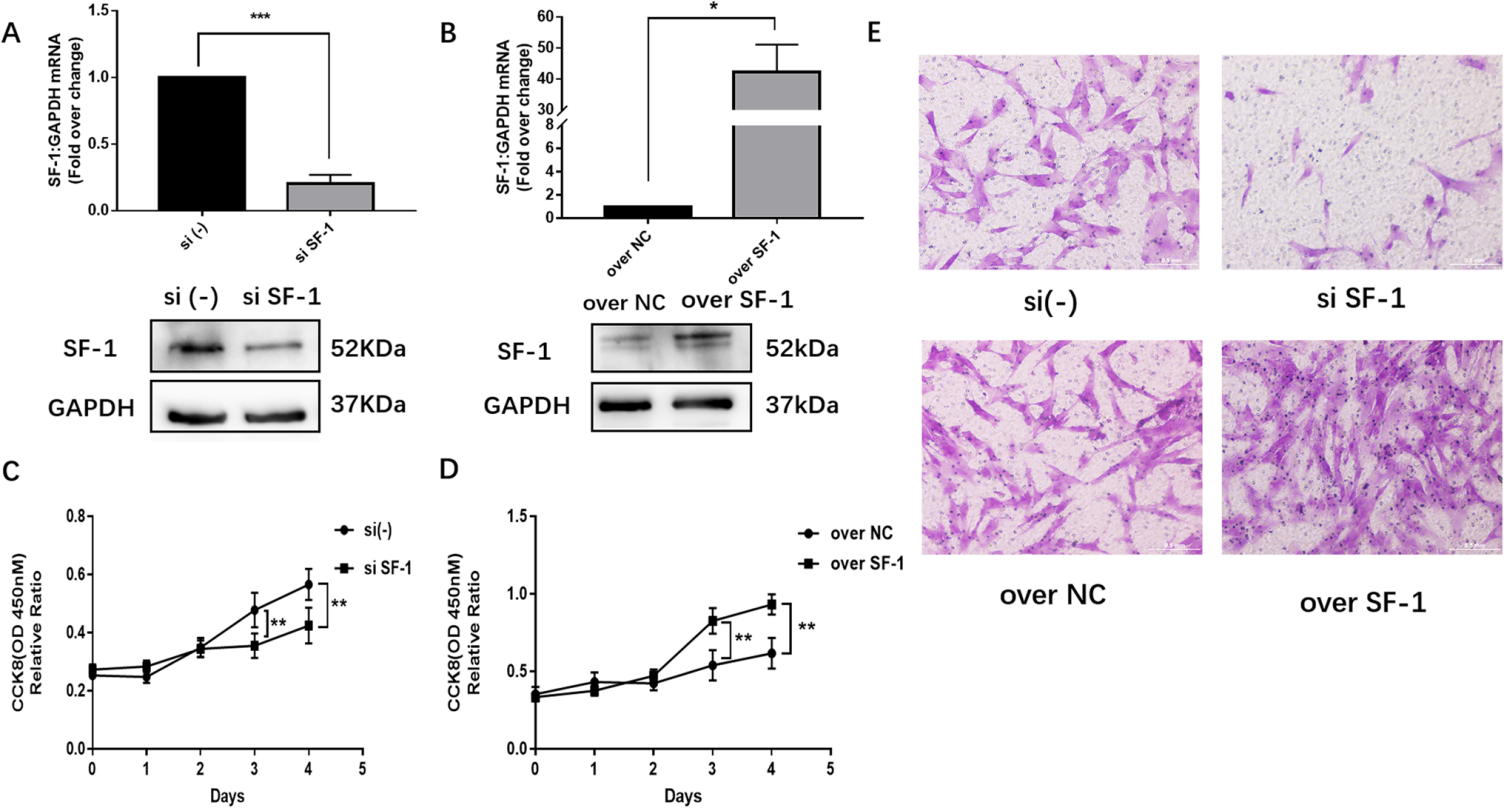
SF-1 promoted the proliferation and invasion of ESCs. (**a**) ESCs were transfected with the indicated siRNAs for 48 h, and the cells were harvested for real-time quantitative PCR or immunoblotting with anti-SF-1 antibody to verify the siRNA knockdown efficiency (*n* = 3; ***, *P* < 0.001, *t*-test). (**b**) ESCs were transfected with the indicated plasmid for 48 h, and the cells were harvested for real-time quantitative PCR or immunoblotting with anti-SF-1 antibody to verify the overexpression efficiency (*n* = 3; ***, *P* < 0.001, *t*-test). (**c** and **d**) ESCs were incubated for 4 days after transfection with the indicated siRNA or plasmid. Cell growth was evaluated using the CCK-8 assay (*n* = 3; **, *P* < 0.01; ANOVA). (**e**) At 48 h post-transfection with the indicated plasmids, the invasion of ESCs was assessed using Matrigel invasion chambers (*n* = 3). Magnification, 40 ×

In an attempt to determine the functional effects of SF-1 on ESCs, we performed CCK-8 and Transwell assays. To knock down or overexpress SF-1, we transfected SF-1 siRNA or SF-1 overexpression plasmid into ESCs. The results of SF-1 knockdown and overexpression in ESCs are presented in Fig. 4a and b. SF-1 knockdown decreased the cell proliferation rate, whereas SF-1 overexpression had the opposite effect on cell proliferation rate (Fig. 4c and d), indicating that SF-1 can promote ESC proliferation. Furthermore, ESCs transfected with SF-1 siRNA showed decreased cell migration, whereas SF-1-overexpressing ESCs showed increased cell migration (Fig. 4e). These results demonstrate that TCF21 can regulate the proliferation and invasion of ESCs.

### SF-1 Reversed the Effects of miR-92a-3p on Cell Proliferation and Invasion

**Fig. 5:**
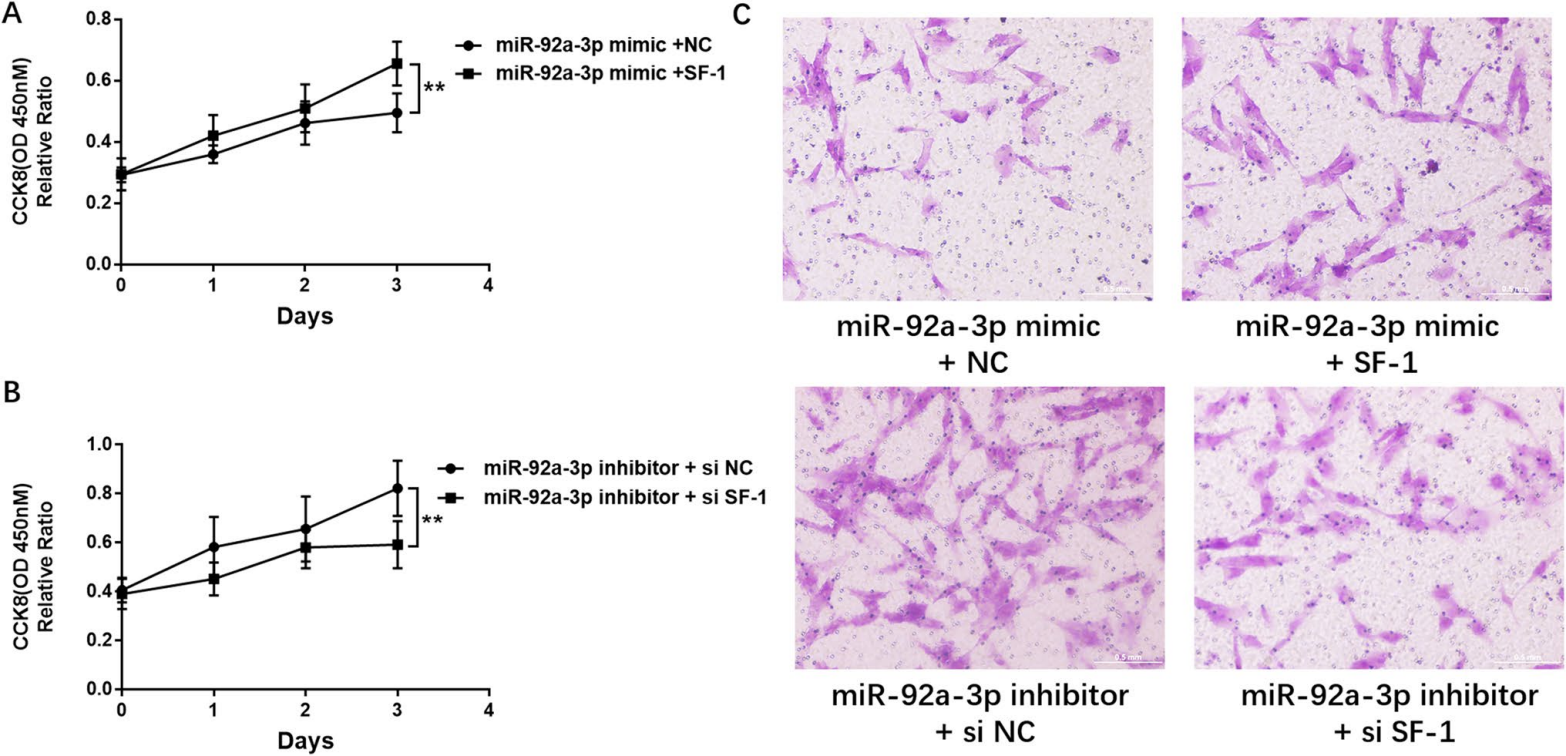
SF-1 reversed the effects of miR-92a-3p on cell proliferation and invasion. (**a** and **b**) ESCs were incubated for 3 days after transfection. Cell growth was evaluated using the CCK-8 assay (*n* = 3; **, *P* < 0.01; ANOVA). (**c**) At 48 h post-transfection with the indicated plasmids, the invasion of ESCs was assessed using Matrigel invasion chambers (*n* = 3). Magnification, 40 ×

To understand the potential mechanism by which miR-92a-3p regulates cell proliferation and migration during EMS, the proliferation and migration of ESCs were assessed after cotransfection with si-SF-1 and the miR-92a-3p inhibitor. The results showed that silencing SF-1 while inhibiting miR-92a-3p partially blocked the enhanced cell proliferation and invasion ability caused by miR-92a-3p knockdown (Fig. 5a). Consistent with these results, overexpression of SF-1 enhanced the decrease in ESC proliferation and invasion induced by miR-92a-3p (Figs. 5b and c). These findings support the hypothesis that miR-92a-3p inhibits the proliferation and invasion of ESCs via SF-1.

## Discussion

EMS is one of the major female health burdens that affects the quality of life and reproductive potential of individuals. To date, the fundamental molecular mechanism of pathogenesis has remained unclear [1]. In recent years, miRNAs have been identified to be involved in various biological processes and in the development and progression of diseases, including EMS [14]. Therefore, identifying EMS-associated miRNAs and their target genes might provide novel therapeutic targets for EMS.

Here, we collected paired eutopic and ectopic endometrium samples during the proliferative phase of the menstrual cycle of patients and used a next-generation sequencing (NGS) platform, which has the advantages of high sensitivity and resolution and excellent reproducibility, to explore the differential expression of miRNAs [19]. The results showed that miR-92a-3p was differentially expressed between ectopic and eutopic endometrium. Numerous studies have demonstrated that miRNA expression is altered in EMS [20–22]. However, due to differences in study design among previous studies, the results have been highly inconsistent. miRNAs are found both intracellularly and in the circulation. Papari E et al. did not find differences in miR-92a-3p levels between EMS and non-EMS samples [23]. The difference in specimens used for sequencing between their study and the present study (plasma vs. tissue) could account for this difference in miRNA expression. Circulating miRNA expression profiles are generally used in the exploration of noninvasive diagnostic biomarkers. In this study, we attempted to investigate the pathogenesis of EMS, so we collected paired EMS tissues for sequencing. In 2009, Ohlsson-Teague and coworkers performed the first assessment of miRNA expression profiles in paired eutopic and ectopic endometrial tissues and identified 14 upregulated and 8 downregulated miRNAs by microarray analysis [24]. However, miR-92a-3p was not found among the differentially expressed miRNAs. The possible reason for this inconsistency between their study and the present study is that there are considerable interplatform differences between NGS, which is the methodology we adopted, and microarrays. Unlike microarrays, NGS provides an unbiased approach to profiling all the transcribed molecules in a sample, as it is not limited to previously known or annotated transcripts [25]. Moreover, NGS accommodates a large dynamic range of expression levels, thereby allowing much more accurate quantification of genes with very low or very high expression levels [26]. In addition to the differences in the specimens used and methodology adopted, a possible reason for the inconsistent results among studies is that miRNA expression in normal endometrium exhibits dynamic changes across the menstrual cycle [27], and previous studies did not clearly define the period of collection of endometrial specimens, which may have affected the study results.

The miR-92a family is a group of highly conserved miRNAs, including miR-25, miR-92a-1, miR-92a-2, and miR-363, which have been reported to be related to tumorigenesis and tumor development [28]. For instance, miR-92a is expressed at a high level in cervical cancer tissues and can promote cell proliferation by inhibiting p21 expression and promoting cell cycle progression [29]. In addition, miR-92a level has been reported to be significantly lower in the tumor tissues of breast cancer patients than in the tissues of healthy controls and to be related to cell migration [30]. These findings suggest that whether miR-92a possesses oncogenic activity depends on the subtype of cells involved. Li et al. [16] found that miR-92a level was markedly increased in patients with progesterone-resistant endometriosis. Moreover, miR-92a has been reported to affect progesterone-resistant EMS by suppressing PTEN expression. However, in the literature, there are no evaluations of miR-92a expression differences between paired eutopic and ectopic endometrium, and the role of miR-92a in the pathogenesis of EMS has not been determined. In this study, we performed in vitro experiments in ESCs. One reason for employing these cells is that EMs or endometriotic stromal cells can be maintained in primary culture to study their biology, whereas this process is more challenging with epithelial cells [1]. Furthermore, several studies have demonstrated that endometriotic stromal cells are epigenetically misprogrammed [1]. Thus, we performed biological experiments with ESCs in this study. Consistent with the NGS results, we found that miR-92a-3p expression in ESCs was lower than that in EMs and confirmed that miR-92a-3p can promote the proliferation and invasion of ESCs.

On the basis of computational algorithms, miR-92a-3p was predicted to regulate the TCF21 gene. A previous study from our group showed that high expression of TCF21 in ESCs could regulate estrogen-signaling pathways and lead to SF-1 overexpression. In the present study, we observed that miR-92a-3p overexpression could reduce TCF21 levels in ESCs transfected with miR-92a-3p mimics, while the opposite effect was observed upon inhibition of miR-92a-3p. These findings are consistent with previous reports that TCF21 is a direct target of miR-92a-3p in osteosarcoma. The results of previous luciferase experiments indicated that miR-92a-3p could directly bind to the 3’-UTR of TCF21 [31]. In this study, we next explored the effect of miR-92a-3p on the expression of SF-1, which has been validated as a downstream gene of TCF21. Consistent with the TCF21 findings, the expression of SF-1 was indirectly regulated by miR-92a-3p. Therefore, the suppression of TCF21 and SF-1 gene expression by the overexpression of miR-92a-3p could potentially be used as a therapy for EMS.

SF-1 is classified as an orphan nuclear receptor because it does not have a well-defined natural ligand. In EMS, SF-1 serves as a key stimulator of estrogen production [32]. In addition to being involved in steroidogenesis, SF-1 is involved in other physiological behaviors of cell function. Several in vivo studies have demonstrated that SF-1 gene expression affects the size of mouse adrenal glands [33]. In addition, SF-1 overexpression in human adrenocortical cells has been shown to increase the proliferation rate in vitro and transcript and microarray analyses have revealed increased expression of regulators of cell cycle progression and reduced expression of proapoptotic factors following SF-1 overexpression [8]. Another study found that the expression of an SF-1 target gene encoding the guanine exchange factor VAV2 was a critical mediator of changes in adrenocortical carcinoma cell morphology that promoted invasion in both culture and in vivo models [9]. The above results indirectly illustrate the regulatory effect of SF-1 on invasion. Although the effects of SF-1 on proliferation and invasion have been investigated previously, they have not been examined in EMS. Thus, we investigated whether SF-1 has the same functional roles in EMS. The CCK-8 assay demonstrated that miR-92a-3p upregulation markedly decreased the proliferation abilities of ESCs. Furthermore, the Transwell invasion assays showed that forced expression of miR-92a-3p significantly reduced the invasive activities of ESCs. It can be inferred that reduced SF-1 expression could result in excessive proliferation and invasion of ESCs and promote EMS development. In addition, the results of the rescue experiments and functional assays demonstrated that restoration of SF-1 expression partly abolished the inhibitory effects of miR-92a-3p on proliferation and migration in ESCs. Moreover, knockdown of SF-1 reversed the increased proliferation and migration in ESCs mediated by miR-92a-3p overexpression. These results suggest that miR-92a-3p regulates the proliferation and migration of ESCs in an SF-1-dependent manner.

Available medical therapies for EMS include nonhormonal treatments, such as pain relievers, and hormonal treatments, such as combined oral contraceptives (COCs), progestins, and gonadotropin-releasing hormone analogs (GnRHa) [34]. However, these treatments have side effects, such as bone loss and inhibition of ovulation [34]. The limitations of currently available options prompted us to seek novel therapies for EMS. In recent years, miRNA technologies have entered the clinical testing stage and might be utilized in novel, nonhormonal therapeutic approaches for EMS [35]. For example, MRG-201, which mimics the activity of miR-29, has been developed by miRagen Therapeutics as a substitute for miR-29. A phase II trial (NCT03601052) is ongoing to study the safety and tolerability of MRG-201 as well as its pharmacokinetics in patients with a history of keloid scars [36]. There have been no clinical trials involving the use of miRNAs in EMS; however, previous studies have demonstrated that treatment with miR-142-3p and miR-200c mimics can decrease the growth of endometriotic lesions in vivo, suggesting the potential therapeutic value of miRNAs in the treatment of EMS [20, 37]. Furthermore, endometriomas have been shown to affect both oocyte production and ovulation in the affected ovary [38]. In previous studies, miR-92a-3p mimic increased antrum formation and diameter; in addition, supplementation with miRNA mimics miR-92a-3p improved the rate of chromatin configuration and the developmental ability of oocytes to the blastocyst stage [39]. These observations suggest that miR-92a-3p cannot only suppress endometriotic graft growth and progression but also improve the oocyte quality of EMS patients.

However, some limitations of our study cannot be neglected. The main limitation of this study is the small sample size, so recruiting more participants is important for future studies. Another limitation is the lack of in vivo experiments with an EMS mouse model; such experiments might validate the therapeutic effect of miR-92a-3p in the pathogenesis of EMS. Additionally, the effect of miR-92a-3p on estradiol production warrants investigation.

In conclusion, by using NGS, we found that miR-92a-3p expression was downregulated in EMS. miR-92a-3p mimic transfection resulted in the inhibition of proliferation and invasion in ESCs. We demonstrated that low miR-92a-3p expression is related to elevated expression of TCF21 and SF-1, which might be involved in the regulation of invasive growth and cell proliferation during EMS development. In addition, our findings revealed that SF-1 could reverse the effect of miR-92a-3p on ESCs. Further studies on the repertoire of aberrantly expressed miRNAs, miRNA–target mRNA interactions, and the regulation and mechanisms of action of miRNAs may provide a promising therapeutic strategy for EMS.

## Data Availability

The datasets generated and analyzed during the current study are available from the corresponding author on reasonable request.
